# Geometrical Synthesis of Sparse Antenna Arrays Using Compressive Sensing for 5G IoT Applications

**DOI:** 10.3390/s20020350

**Published:** 2020-01-08

**Authors:** Giulia Buttazzoni, Fulvio Babich, Francesca Vatta, Massimiliano Comisso

**Affiliations:** Department of Engineering and Architecture, University of Trieste, Via A. Valerio 10, 34127 Trieste, Italy; babich@units.it (F.B.); vatta@units.it (F.V.); mcomisso@units.it (M.C.)

**Keywords:** antenna arrays, sparse arrays, geometrical synthesis, power pattern synthesis, compressive sensing

## Abstract

One of the main targets of the forthcoming fifth-generation (5G) cellular network will be the support of the communications for billions of sensors and actuators, so as to finally realize the Internet of things (IoT) paradigm. This pervasive scenario unavoidably requires the design of cheap antenna systems with beamforming capabilities for compensating the strong attenuations that characterize the millimeter-wave (mmWave) channel. To address this issue, this paper proposes an iterative algorithm for sparse antenna arrays that enables to derive the number of elements, their amplitudes, phases, and positions in the presence of constraints on the far-field pattern. The algorithm, which relies on the compressive sensing approach, is formulated by transforming the original nonconvex optimization problem into a convex one. To prove the suitability of the conceived solution for 5G IoT mmWave applications, numerical examples and comparisons with other existing methods are provided, considering synthesis problems with different pattern and aperture specifications.

## 1. Introduction

The idea of combining the radiation of multiple antennas to obtain specific pattern shape dates back to the beginning of the 19th century, when the history of wireless communications began [1,2]. Nowadays, antenna arrays are widely used in a large variety of scenarios involving far- and near-field focusing/multifocusing applications for satellite, cellular, vehicular, and sensor networks [3,4,5,6,7,8,9,10,11,12,13,14,15,16,17]. However, even if the array technology is already widespread, its importance is expected to further increase, since the directionality of the communications will represent a basic enabling functionality of the forthcoming fifth-generation (5G) and Internet of things (IoT) systems [18,19,20,21]. This forecast is motivated, on one hand, by the expected presence of a huge number of active devices (smartphones, sensors, actuators), and, on the other hand, by the adoption of the millimeter-wave (mmWave) bands. In fact, the need of attenuating the reciprocal interference among the 5G equipments and of compensating the significant attenuations that characterize the mmWave channel implies the implementation of multi-antenna systems satisfying compactness and performance constraints. These constraints must be of course combined with the possible reduction in the unit price of a device, so as to better match the market demand. An initial way to achieve this requirement is to minimize the number of radiators in each antenna array. However, due to the so crowded electromagnetic environment, this task cannot be accomplished at the expense of the desired radiation characteristics. Therefore, the array synthesis problem addressed by a 5G antenna designer is very hard to solve, since it requires to optimize not only the excitations of the array elements, but also their positions and, possibly, their number. This situation identifies the typical problem addressed in the research field represented by the design of sparse antenna arrays [22].

This latter issue has attracted the interests of the research community since the 1960s [23,24,25,26,27], regaining attention in the more recent years thanks to its applicability to the emerging communication scenarios. Accordingly, several methods have been proposed in the literature for the synthesis of sparse antenna arrays by considering both stochastic and deterministic approaches [28,29,30,31,32,33,34,35,36,37,38,39,40,41,42,43]. Stochastic methods can rely on different strategies, including genetic algorithms [28,29], particle swarm optimization [30], differential evolution [31], and nature inspired techniques, such as the ant [32], whale [33], and grey wolf [34] optimization algorithms. Also deterministic methods have been developed by moving from different strategies. In particular, a noniterative algorithm is proposed in [35], where the matrix pencil method is adopted to approximate a desired pattern using a nonuniform linear antenna array with a reduced number of elements. In [36], the same authors present a deterministic three-step procedure able to optimize the magnitudes, phases, and locations of the elements of a linear antenna array radiating a shaped power pattern. In [37], the alternating projection algorithm is applied in conjunction with a special technique, which iteratively places the radiating elements with the aim of synthesizing a sparse array starting from a power pattern mask specification. In [38], a low-complexity deterministic method for the synthesis of phase-only beam scanning linear aperiodic arrays is proposed, which optimizes the positions, the amplitudes, and the phases of the array elements. Convex optimization techniques are instead applied to the synthesis of sparse antenna arrays in [39,40,41,42,43], where, the compressive sensing (CS) strategy is employed. The CS technique was originally introduced in the field of image processing and signal reconstruction [44], but nowadays its applications have hugely spanned. Thus, many different types of engineering problems have been solved by the CS approach, some of which specifically belongs to the electromagnetics’ research context, such as the diagnosis and synthesis of antenna arrays, the estimation of the direction of arrivals, and the solution of inverse scattering and radar imaging problems [45]. With particular reference to the synthesis of sparse arrays, the main advantage of CS consists in the possibility to convert a nonconvex formulation into a convex one, thus facing a complex problem in an acceptable processing time.

According to the above observations, this paper considers the synthesis of sparse antenna arrays for mmWave applications by developing an iterative algorithm based on the CS optimization. The algorithm is conceived to address the general case in which a fixed grid structure is not available, and so nonlinear problems characterized by complex formulations have to be managed, even when simple array geometries and not challenging radiation constraints are involved. In fact, the aim is to enable the 5G antenna designer to synthesize not only the array excitations (in amplitude and phase), but also the number and the location of the elements in the presence of requirements on the far-field pattern.

In this sense, the main advantage of the presented method with respect to the previously proposed CS-based ones, consists in the possibility to impose that the synthesized pattern belongs to a predefined mask. Differently from the conventional pattern matching approach, where a specific shape is imposed both inside the main lobe (region of interest) and outside it (region not of interest), the here proposed algorithm applies in detail the shape constraint to the sole main lobe, imposing just an upper limit to the other angular regions. Moreover, the main lobe shaping is realized independently of the pattern phase. Thus, a power pattern problem is here addressed instead of a field synthesis one analyzed in [40]. In this way, the available degrees of freedom are better exploited. Besides, the pattern requirements are matched by reformulating the initial nonconvex problem into a convex one in order to enable its mathematical tractability in an acceptable CPU time. To prove the effectiveness of the presented method, numerical examples adherent to the 5G IoT context and comparisons with existing approaches are presented and discussed.

The remaining of the paper is organized as follows. Section 2 formulates the addressed problem. Section 3 presents the developed algorithm. Section 4 discusses the numerical results. Section 5 summarizes the most relevant conclusions.

*Notation*. Throughout the paper the following notation is used: ${\left(\cdot \right)}^{T}$ denotes the transpose operator, ${\left(\cdot \right)}^{*}$ denotes the complex conjugate, *j* denotes the imaginary unit, and ∠x denotes the argument of *x*.

## 2. Problem Formulation

With reference to a Cartesian system O(x,y,z), consider an antenna array of arbitrary geometry composed by a generic (and possibly large) number of elements *N*, which are located at the positions ${\bar{r}}_{n}=x_{n}\hat{x}+y_{n}\hat{y}+z_{n}\hat{z}$, for n=1,…,N, where $\hat{x},\hat{y}$ and $\hat{z}$ represent the unit vectors of the corresponding Cartesian axes. As usual, in spherical coordinates, denote by θ the angle measured from the *z*-axis and by ϕ the azimuth angle. The radiation pattern produced by this arbitrary array at the generic direction $\hat{r}=\sin\theta \cos\phi \;\hat{x}+\sin\theta \sin\phi \;\hat{y}+\cos\theta \;\hat{z}$ is given by:

(1) $$F\left(w;\hat{r}\right)=\sum_{n=1}^{N}w_{n}\;f_{n}\left(\hat{r}\right)\exp\left(jk\;{\bar{r}}_{n}\cdot \hat{r}\right),$$

where $w={\left[w_{1},\ldots ,w_{N}\right]}^{T}$ represents the column vector of the complex excitations of the array elements, $f_{n}\left(\hat{r}\right)$ is the element pattern of the *n*-th array element, and k=2π/λ is the wavenumber, being λ the carrier wavelength.

Concerning (1), two main aspects should be taken into account. Firstly, the geometry of the array, which may be linear, planar or even conformal, is usually specified by some shape/size constraints in the physical design. When the CS strategy is adopted, the application of these constraints allows one to identify the possible positions of the *N* elements as the candidate positions, while the final array will be composed by a very reduced number of elements, suitably chosen among these *N* candidates. The second aspect that should be considered is that also the pattern generated by (1) is always required to satisfy specific shape constraints, which can be properly modeled by a suitably defined mask. Accordingly, the constraints on the pattern can, in principle, be expressed as:

(2) $$M^{\mathrm{low}}\left(\hat{r}\right)\leq \left|F\left(w;\hat{r}\right)\right|\leq M^{\mathrm{up}}\left(\hat{r}\right),$$

where $M^{\mathrm{low}}\left(\hat{r}\right)$ and $M^{\mathrm{up}}\left(\hat{r}\right)$ are real positive functions representing, respectively, the lower and upper bound of the mask.

The problem addressed in this paper is that of selecting, among the *N* candidate positions, the lowest number of array elements, their positions and excitations in such a way as to obtain a radiation pattern compliant with (2). By consequence, the here addressed problem can be mathematically formulated in compact form as follows:

(3a) $$\begin{array}{cc} \; & \min_{{\begin{array}{c} w\in C^{N^{{}^{{}^{}}}} \end{array}}_{{}_{{}_{{}_{{}_{{}_{{}_{}}}}}}}\;\;\;\;}{∥w∥}_{0} \end{array}$$

(3b) $$\begin{array}{cc} \; & \mathrm{subject}\;\mathrm{to}\;(2) \end{array}$$

where ${∥w∥}_{0}$ denotes the $l_{0}$-norm of w. More precisely, ${∥w∥}_{0}$ counts the nonzero components of w, corresponding to the elements that will result physically active at the end of the synthesis process.

## 3. Synthesis Method

As it can be immediately observed, the problem in (3) is in general nonlinear and nonconvex, thus extremely hard to solve. The CS approach is specifically useful for this kind of situations, but requires a proper reformulation of (3). To this aim, consider an iterative procedure, which, at the generic *k*-th iteration, solves a minimization problem whose objective function is given by the weighted $l_{1}$-norm (or weighted Manhattan distance) [39]:

(4) $$∥w^{k}{∥}_{1}=\sum_{n=1}^{N}\alpha_{n}^{k}\left|w_{n}^{k}\right|,$$

in which, for n=1,…,N, k=1,2,…, the weights:

(5) $$\alpha_{n}^{k}=\left(\right|w_{n}^{k-1}{\left|+\varepsilon \right)}^{-1},$$

are introduced to improve the final result by properly selecting a suitable parameter ε. The objective function in (4) along with the weight definition in (5) replaces the original objective function in (3a), and is known to produce sparse solutions [46]. However, this novel problem remains difficult to solve because of the nonlinearity and nonconvexity of (2)–(3b). To address this second aspect, let outline some observations regarding the radiation pattern requirements that are commonly imposed. Usually, a desired pattern is characterized by: (i) a main beam region (MBR) with a shape that must be exactly matched, (ii) a maximum allowed level for the sidelobe region (SLR), and (iii) a (possible) null region (NR). For these two latter requirements, the lower bound of the mask is not of concern, since the lower is the pattern, the more appreciable is the result. Therefore, according to [40], a suitable strategy to handle the constraint in (2)–(3b) is to separately impose the requirement on the MBR and those on the SLR and NR. Accordingly, (2) is subdivided into the following two constraints:

{(6a)|F(w;r^)−Fd(r^)|≤δr^∈MBR(6b)|F(w;r^)|≤Mup(r^)r^∈SLR∪NR

where $F_{d}\left(\hat{r}\right)$ represents the desired main lobe function [40]. Now, it is worth to note that the usage of (6a) leads to a field synthesis problem and not to a power synthesis one. This implies that the available degrees of freedom are in part wasted to approximate the array phase pattern, which is usually not of interest. To overcome this issue, one can first recall the following property holding for two arbitrary complex numbers $z_{1}$ and $z_{2}$:

(7) $$\left|\right|z_{1}\left|-\right|z_{2}\left||=\right|z_{1}-z_{2}|\;\mathrm{iff}\;∠z_{1}=∠z_{2}+2h\pi ,\;h\in Z.$$

This property suggests that (6a) is equivalent to a power synthesis problem if and only if the phase of the array pattern equals the phase of the desired pattern. Hence, by considering (4)–(7), the original problem in (3) can be iteratively solved by minimizing, at the *k*-th iteration, the constrained function:

(8a) $$\begin{array}{cc} \; & \min_{{\begin{array}{c} w^{k}\in C^{N^{{}^{{}^{}}}} \end{array}}_{{}_{{}_{{}_{{}_{{}_{{}_{}}}}}}}\;\;\;\;}{∥w^{k}∥}_{1} \end{array}$$

(8b)subjectto:|F(wk;r^)-Fdk(r^)|≤δ(r^)r^∈MBR(8c)|F(wk;r^)|≤Mup(r^)r^∈SLR∪NR

where $\delta (\hat{r})$ is a real function defining the accuracy required in the MBR at each direction, and:

(9) $$F_{d}^{k}\left(\hat{r}\right)=F_{d}^{0}\left(\hat{r}\right)\exp\left[j∠F(w^{k-1};\hat{r})\right],$$

is the desired MBR function updated, at the *k*-th iteration, according to the phase of the pattern synthesized at the (k-1)-th iteration and to a suitable real function $F_{d}^{0}\left(\hat{r}\right)$ defining the MBR shape. Notably, this latter formulation represents a second-order cone program (SOCP) problem, which can be solved by the use of freely available software routines, as, for example, the Matlab-based CVX [47]. It is also worth to observe that, although, at each iteration, (8a)–(8c) formally belongs to the class of field synthesis problems, the iterative modification in (9) of the constraint in (8b) actually leads to a power synthesis problem. Importantly, note that it is this iterative modification of the MBR constraint that allows one to better exploit the degrees of freedom of the problem and thus to improve the final results. Moreover, to refer all requirements in terms of mask specifications, one can define the functions identifying the accuracy and the MBR shape, respectively, as:

(10a) $$\begin{array}{cc} \; & \delta \left(\hat{r}\right)=\frac{M^{\mathrm{up}}\left(\hat{r}\right)-M^{\mathrm{low}}\left(\hat{r}\right)}{2}, \end{array}$$

(10b) $$\begin{array}{cc} \; & F_{d}^{0}\left(\hat{r}\right)=\frac{M^{\mathrm{up}}\left(\hat{r}\right)+M^{\mathrm{low}}\left(\hat{r}\right)}{2}. \end{array}$$

This operation enables to finally identify the iterative algorithm that solves the original synthesis problem formulated in (3). The development of the algorithm is detailed in Table 1. Step 1 specifies the problem’s constraints (candidate positions, bounds of the mask, accuracy, SLR, NR, and initial MBR shape). Step 2 initializes the iteration and the excitations. Step 3 updates the iteration, the pattern, the weights, and the phase of the MBR shape. This latter update constitutes the major novelty of the proposed approach. Step 4 solves the SOCP problem at the present iteration. Step 5 identifies the stop condition, which requires that the number of nonzero elements of the excitation vector does not change in three consecutive iterations. Alternatively, the stop condition might also be formulated by selecting a maximum pre-defined number of iterations. Of course, during the evolution of the algorithm, none element of $w^{k}$ does exactly vanish, but the generic *n*-th element of the array is considered as zero, that is, is assumed absent, if the amplitude of its excitation $|w_{n}^{k}|$ is lower than the threshold ε.

## 4. Results and Discussion

Five numerical examples are provided to prove the effectiveness of the proposed algorithm, by considering both two-dimensional (2D) problems (i.e., azimuth synthesis only) and three-dimensional (3D) ones (i.e., zenith-azimuth synthesis). Four of these examples are taken from the literature, in order to have a direct benchmark with the state-of-the-art methods in terms of element saving, while the fifth example is specifically conceived to include a more evolved multi-ring array geometry. Note that the first four problems are intentionally selected from examples already proposed in previous papers, since, according to the approach commonly adopted in the array synthesis research field, a direct comparison between the proposed and the existing algorithms can be immediately carried out. Furthermore, to put into evidence the relevance of all the solved problems for the IoT scenario, each example is described including specific references in which the employed array is exploited for 5G sensor applications. The algorithm is implemented using Matlab R2018b and CVX on a personal laptop having 8 GB RAM, and all results are obtained adopting a threshold $\varepsilon =10^{-2}$ [40].

### 4.1. First Example

The first numerical example considers the 2D problem addressed in [39], which involves a linear array of isotropic elements lying on the *z*-axis and characterized by an aperture equal to 20λ. This array is required to radiate in the zenith domain a flat-top pattern, in which the MBR is given by the set $\Theta_{\mathrm{MBR}}=\left\{\theta :70^{\circ }\leq \theta \leq 110^{\circ }\right\}$ with an allowed ripple $\rho_{t}$ equal to 0.4455 dB, while the SLR is defined by the set $\Theta_{\mathrm{SLR}}=\left\{\theta :0^{\circ }\leq \theta \leq 65^{\circ }\cup \;115^{\circ }\leq \theta \leq 180^{\circ }\right\}$ with a maximum sidelobe level equal to -30 dB. The initial set of candidate positions is selected by regularly spacing the isotropic radiators on the available aperture with an inter-element distance of λ/100, resulting in a starting value N=2001. The linear array geometry has been widely applied to IoT scenarios [19,20,48], thanks to its simplicity of realization and conformability to objects having the length as the prevailing dimension.

**Figure 1:**
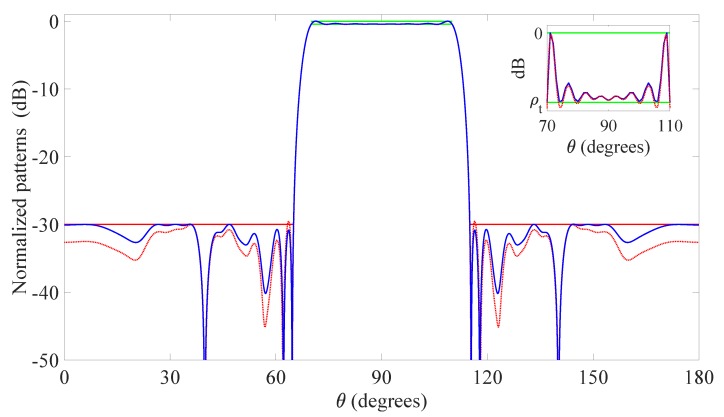
First example: linear array. Flat-top pattern radiated by the optimized 19 elements (blue solid line), upper and lower bounds of the mask in the main beam region (MBR) (green solid line), upper bound of the mask in the sidelobe region (SLR) (red solid line), pattern synthesized by the 17 elements obtained after minimum inter-element distance control (red dashed line). The inset shows a zoom of the MBR. The final positions and excitations are listed in Table 2.

The pattern obtained using the proposed algorithm is reported in Figure 1. Table 2, instead, lists the element positions normalized with respect to λ and the excitations normalized with respect to $w_{10}$. From these values one may first observe that, being the desired pattern symmetrical, the active elements are symmetrically displaced and the corresponding excitations are real numbers. Of course, the central element n=10 lies in position $z_{10}=0$ and has a normalized excitation equal to one. Interestingly, among the N=2001 candidates, just 19 elements have been sufficient to match the pattern requirements. This represents a considerable reduction of the final number of elements as compared to [39], in which the final array was composed by 31 elements, a result already better than the 41 elements calculated in [49,50], where this problem was originally developed.

A possible further reduction of the number of elements might be achieved by imposing a further minimum inter-element distance control, which in this case can be considered suitable. In fact, one may notice from Table 2 that the elements n=8 and n=9, and, similarly, the elements n=11 and n=12, are very close to each other. In particular, their distance is exactly equal to the grid step λ/100, thus making difficult the practical realization of the array. The proposed refinement consists in replacing the pairs (8,9) and (11,12) with two single elements lying in the middle points $(z_{8}\;+\;z_{9})/2\;=\;-0.645\lambda$ and $(z_{11}\;+\;z_{12})/2\;=\;0.645\lambda$, and selecting, for the respective excitations, the values $w_{8}\;+\;w_{9}\;=\;0.6351$ and $w_{11}\;+\;w_{12}\;=\;0.6341$. In this way, one can obtain an array of only 17 elements that radiates the pattern in Figure 1 identified by the red dashed line, which is characterized by a very limited degradation with respect to the originally synthesized one (blue solid line). As a final observation, it is worth to note that the here derived original pattern has been obtained after six iterations that have required 254 s. Thus, just a few minutes have been sufficient to achieve the presented result.

### 4.2. Second Example

The second example is still a 2D flat-top synthesis problem taken from [39]. The problem considers the same linear array and candidate elements of the first example, but, differently, the array is composed by directive radiators, which are more likely to be mounted on actual IoT sensors [19,20,48]. More precisely, the single-element pattern is modeled by the function $f_{n}\left(\theta \right)=\sin\theta$, representing a short dipole parallel to the *z*-axis. Moreover, the desired MBR is no more symmetrical with respect to the broadside direction, but is defined by the shifted set $\Theta_{\mathrm{MBR}}=\left\{\theta :50^{\circ }\leq \theta \leq 90^{\circ }\right\}$ with an allowed ripple $\rho_{t}$ equal to 1 dB, while the SLR is defined by the set $\Theta_{\mathrm{SLR}}=\left\{\theta :0^{\circ }\leq \theta \leq 43^{\circ }\cup \;97^{\circ }\leq \theta \leq 180^{\circ }\right\}$ with a maximum sidelobe level equal to -30 dB.

**Figure 2:**
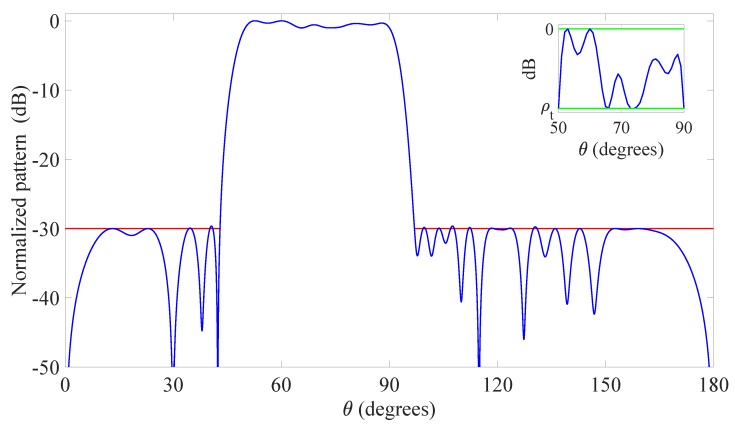
Second example: Linear array. Flat-top pattern radiated by the optimized 18 elements (blue solid line), desired pattern in the MBR (green solid line), upper bound of the mask in the SLR (red solid line). The inset shows a zoom of the MBR. The final positions and excitations are listed in Table 3.

The pattern derived using the developed method is shown in Figure 2, while Table 3 reports the normalized element positions and the complex excitations. In this second example, the desired pattern is not symmetrical, and hence the positions are also not symmetrical and the excitations are not real. The provided results confirm the satisfactory behavior of the proposed technique, since the final array, derived after 13 iterations that have required a CPU time of 548 s (less than 10 min), consists of just 18 active elements. A significant improvement as compared to the 25 elements obtained in [39]. Beside the element reduction, this example proves that the presented method is suitable not only when broadside patterns and isotropic sources are assumed, but also when more general scenarios, characterized by steered main beams and directive radiators, have to be realistically managed.

### 4.3. Third Example

The third example involves the 2D synthesis problem originally developed in [51] that adopts a linear array of aperture 7.5λ composed by isotropic elements. In this case, the algorithm is required to generate a not symmetrical cosecant-like pattern in the MBR defined by the set $\Theta_{\mathrm{MBR}}=\left\{\theta :98^{\circ }\leq \theta \leq 135^{\circ }\right\}$, and to impose a NR given by the set $\Theta_{\mathrm{NR}}=\left\{\theta :66^{\circ }\leq \theta \leq 88^{\circ }\right\}$ in which no more than -30 dB of radiation are allowed. Besides, the SLR is defined by the set $\Theta_{\mathrm{SLR}}=\left\{\theta :0^{\circ }\leq \theta \leq 66^{\circ }\cup \;143^{\circ }\leq \theta \leq 180^{\circ }\right\}$ with a maximum sidelobe level equal to -20 dB, and the pattern obtained in [36] for the same example is used as the desired pattern in (9) in the MBR. Also in this case, the candidate positions are chosen by regularly spacing the radiators on the available aperture with an inter-element distance equal to λ/100, resulting now in a starting value N=751. Note that, with reference to forthcoming pervasive communication networks, the cosecant-like pattern can be of specific interest for IoT sensors, since it enables to radiate a constant power on a given angular region, thus realizing a uniform covering of that region for monitoring applications [52,53].

**Figure 3:**
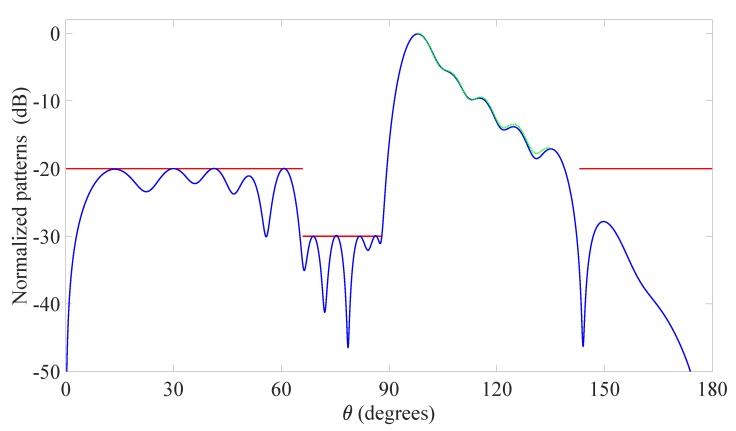
Third example: linear array. Cosecant-like pattern radiated by the optimized 12 elements (blue solid line), desired pattern in the MBR (green solid line), upper bound of the mask in the SLR and null region (NR) (red solid line). The final positions and excitations are listed in Table 4.

The pattern calculated employing the presented approach is reported in Figure 3, while Table 4 shows the normalized element positions and the complex excitations. These results reveal the capability of the developed algorithm to strictly approximate the desired pattern, even in the presence of a wide null region and of a main beam requiring a specific not-flat shape. However, the main advantage of the designed method remains the low number of resulting active elements as compared to the previously proposed techniques. In fact, the original solution in [51] was characterized by 16 elements, and those in [36] and [40] by 13 elements, while the here conceived algorithm has provided, after just four iterations, a final array consisting of only 12 active elements. Furthermore, the time necessary to achieve this result has been approximately equal to 55 s, thus lower than a minute.

### 4.4. Fourth Example

The fourth example is a 3D zenith-azimuth synthesis problem originally developed in [39] that involves a square array of isotropic radiators lying in the x-y plane and having side equal to 5λ. The mask specifications are given in terms of the variables u=sinθcosϕ and v=sinθsinϕ. In particular, the MBR, in which the allowed ripple $\rho_{t}$ is equal 1 dB, is defined by the 2D set $\Omega_{\mathrm{MBR}}=\left\{\left(u,v\right):u^{2}+v^{2}\leq 1/25\right\}$ while the SLR, in which the maximum allowed level is equal to -25.85 dB, is identified by the 2D set $\Omega_{\mathrm{SLR}}=\left\{\left(u,v\right):u^{2}+v^{2}\geq 4/25\right\}$. The initial set of the candidate positions is composed by a regular grid of elements spaced by λ/4 in both directions and covering the available aperture. This leads to a starting value N=441. This fourth example is of interest both for 5G base stations (BSs) and gigabit-WiFi access points (APs) [54], which are, on one hand, characterized by a planar structure that enables the possibility to host many radiating elements [55,56,57], and, on the other hand, have to manage the problem of initial user access, which requires a wide main beam to avoid too long searching procedures for identifying the region of space where a given user lies [58].

**Figure 4:**
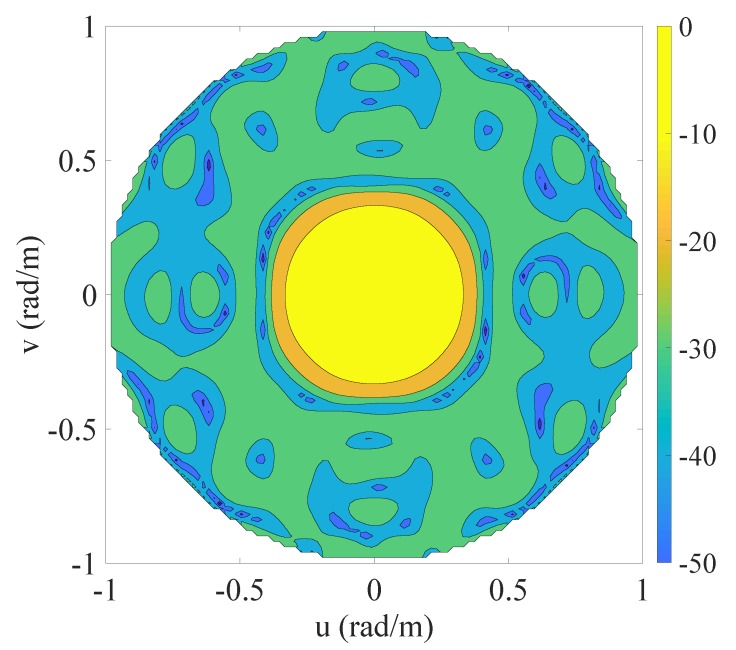
Fourth example: planar array. Contour plot of the synthesized pattern radiated by the optimized 60 elements. The final positions are shown in Figure 5 and the excitations are listed in Table 5.

**Figure 5:**
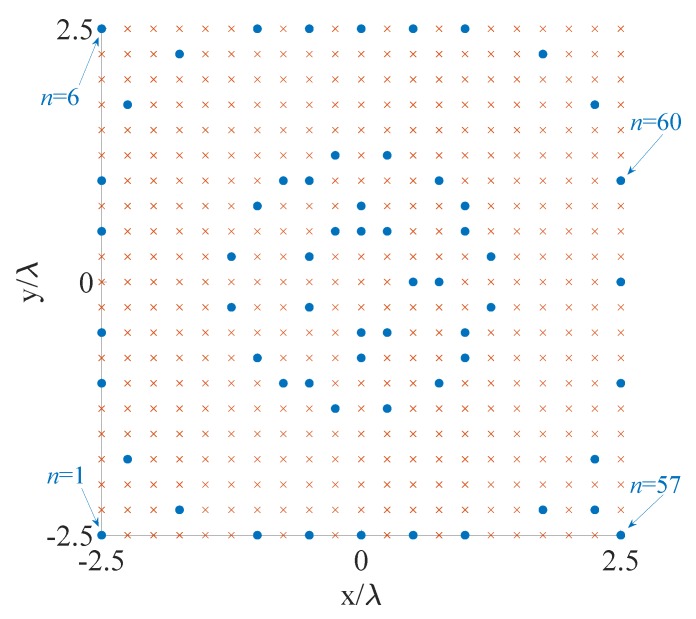
Fourth example: positions of the candidate (red cross) and final (blue circles) array elements.

The contour plot of the pattern derived using the proposed algorithm is shown in Figure 4, while Figure 5 and Table 5 report the initial grid (red cross) with the finally active elements (blue circles) and the real excitations, respectively. For the correspondence between Figure 5 and Table 5, the active elements have been numbered from the bottom to the top and from the left to the right. For readability reasons, Figure 5 illustrates the sole four elements that enable the reader to infer the order. From these results, one may notice that 60 elements have been obtained. A considerable reduction as compared to the 76 derived in [39]. The computational time required to achieve the here provided solution is equal to 2032 s, corresponding to 13 iterations.

### 4.5. Fifth Example

The fifth and last example is a 3D zenith-azimuth synthesis problem developed adopting a planar circular array with multiple rings radiating a very narrow beam. The multi-ring array, having a maximum radius equal to 10λ, consists of isotropic radiators lying in the x-y plane. The initial set of the candidate positions is composed by N=1308 elements regularly spaced on 20 rings having center in the origin of the reference system and radii $R_{i}=0.5\left(1+i\right)\lambda ,\;i=0,\ldots ,19$, in such a way as $d_{\min}\geq \lambda /2$. The mask specifications, given in terms of the variables *u* and *v*, have the MBR defined by the 2D set $\Omega_{\mathrm{MBR}}=\left\{\left(u,v\right):u^{2}+v^{2}\leq 1/400\right\}$ and the SLR identified by the 2D set $\Omega_{\mathrm{SLR}}=\left\{\left(u,v\right):u^{2}+v^{2}\geq 1/100\right\}$. The allowed ripple in the MBR is $\rho_{t}=1$ dB and the maximum allowed level in the SLR is equal to -15 dB. This example refers to a scenario in which the 5G BS or the gigabit-WiFi AP (both suitable to host planar array structures), has already accomplished the initial access with the user and intends to generate a highly directional communication for realizing a high-capacity link [38,59,60].

**Figure 6:**
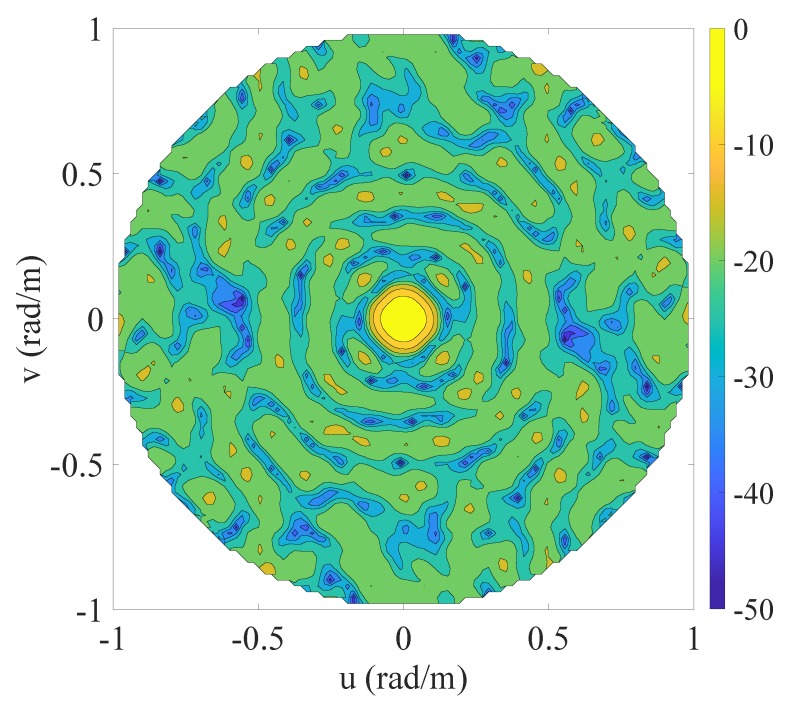
Fifth example: multi-ring array. Contour plot of the synthesized pattern radiated by the optimized 62 elements. The final positions are shown in Figure 7 and the excitations are listed in Table 6.

**Figure 7:**
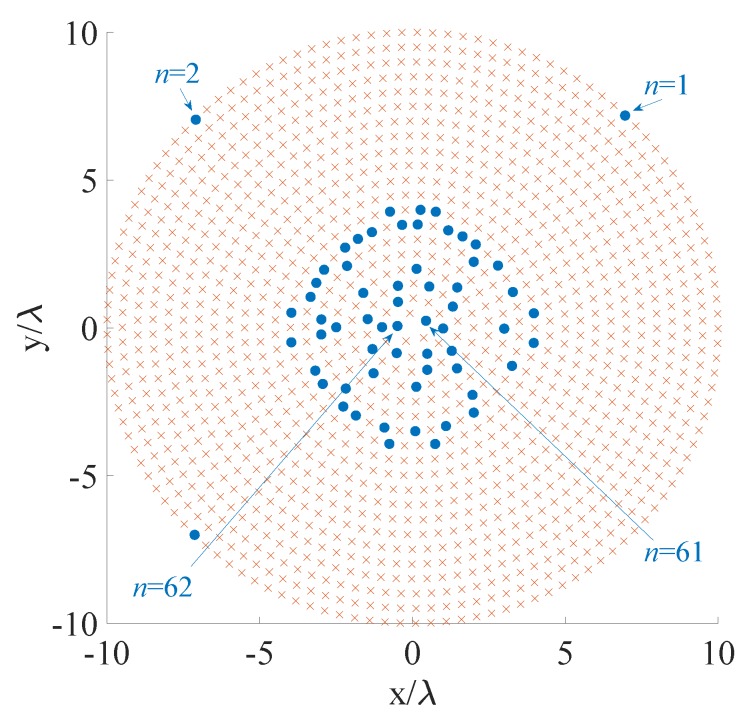
Fifth example: positions of the candidate (red cross) and final (blue circles) array elements.

The contour plot of the pattern derived using the proposed algorithm is shown in Figure 6, while Figure 7 and Table 6 report the initial grid (red cross) with the finally active elements (blue circles) and the real excitations, respectively. For the correspondence between Figure 7 and Table 6, the active elements have been numbered starting from the x- axis counterclockwise from the outermost to the innermost ring. For readability reasons, Figure 7 illustrates the numbers of the four elements that enable the reader to infer the order. The computational time required to achieve the here provided solution is higher with respect to the previous example (14,691 s, corresponding to eight iterations). This is due to the finer grid required to sample a so narrow beam in the u-v domain. This last example and the previous one prove that also 3D synthesis problems may be successfully dealt with by the conceived CS-based approach, considering both flat-top and pencil beam pattern requirements.

A final comment regarding the presented results concerns the mutual coupling effects, which, in the research field covered by the geometrical synthesis of antenna arrays, must be considered once the final positions have been estimated. In fact, the initial set of positions does not represent real elements, but only candidate ones. Hence, when the selected elements change, also the coupling effects change. Consequently, one should evaluate the coupling at each iteration. Clearly, this is not a practical approach and hence the coupling effects may be considered at the end of the procedure. In this case, if the pattern distortion is not acceptable, the element excitations may be modified with any suitable algorithm for the synthesis of conformal arrays, as for example [49,50,61,62,63,64].

## 5. Conclusions

A CS-based iterative procedure for the power synthesis of sparse arrays has been presented. The proposed algorithm relies on the solution of a sequence of SOCP problems with the aim of minimizing the number of radiators of an array composed by a large number of candidate elements. The constraints of the minimization problem have been formulated in such a way as to be convex, and to approximate a power pattern synthesis problem in the entire visible region. The constraints formulation, and, in particular, their iterative modification constitutes the original contribution, which allows one to improve the performance of the previously proposed CS-strategies for sparse array applications. Different numerical examples involving linear and planar structures have been discussed, obtaining, in all cases, the matching of the pattern requirements, and, for the cases involving the comparison with the previous existing solutions, a lower final number of active elements.

## Figures and Tables

**Table 1:** Proposed algorithm.

Step 1	**Problem specifications** (i) Initial set of candidate positions ${\bar{r}}_{n}$ (n=1,…,N); (ii) Upper $M^{\mathrm{up}}\left(\hat{r}\right)$ and lower $M^{\mathrm{low}}\left(\hat{r}\right)$ bounds of the mask; (iii) MBR, SLR, and NR; (iv) Accuracy $\delta (\hat{r})$ and MBR shape $F_{d}^{0}\left(\hat{r}\right)$ using (10a) and (10b), respectively.
Step 2	**Initializations** (i) Iteration k=0; (ii) Excitations $w_{n}^{0}=1$ (n=1,…,N).
Step 3	**Updates** (i) Iteration k→k+1; (ii) Current pattern $F(w^{k-1},\hat{r})$ using (1); (iii) Weights $\alpha_{n}^{k}$ (n=1,…,N) using (5); (iv) Constraint (8a) using (9).
Step 4	**Evaluation** Solve the SOCP problem given by (8a)–(8c).
Step 5	**Stop condition** If k≥3 and $∥w^{k}{∥}_{0}=∥w^{k-1}{∥}_{0}={∥w^{k-2}∥}_{0}$ $w^{k}$ identifies the elements of the final sparse array and their excitations; Exit else Go to Step 3 end

**Table 2:** First example: normalized element positions and excitations.

*n*	$z_{n}/\lambda$	$w_{n}/w_{10}$	*n*	$z_{n}/\lambda$	$w_{n}/w_{10}$	*n*	$z_{n}/\lambda$	$w_{n}/w_{10}$
1	−9.74	−0.0020	7	−1.97	−0.2027	14	3.25	0.1189
2	−8.48	0.0283	8	−0.65	0.1223	15	4.58	−0.0862
3	−7.18	−0.0349	9	−0.64	0.5128	16	5.87	0.0540
4	−5.87	0.0570	11	0.64	0.4826	17	7.18	−0.0399
5	−4.58	−0.0748	12	0.65	0.1515	18	8.48	0.0186
6	−3.25	0.1252	13	1.97	−0.2138	19	9.74	−0.0172

**Table 3:** Second example: normalized element positions and complex excitations.

*n*	$z_{n}/\lambda$	$\left(|w_{n}|\right.,\;\left.∠w_{n}\right)\;$	*n*	$z_{n}/\lambda$	$\left(|w_{n}|\right.,\;\left.∠w_{n}\right)\;$	*n*	$z_{n}/\lambda$	$\left(|w_{n}|\right.,\;\left.∠w_{n}\right)\;$
1	−7.33	(0.0510, −77.4970)	7	−2.25	(0.1816, 110.4115)	13	1.92	(0.2459, −22.6474)
2	−6.01	(0.0065, −34.0712)	8	−1.92	(0.2638, 11.3857)	14	2.25	(0.1407, −120.9294)
3	−4.93	(0.1053, 50.8963)	9	−0.73	(0.5726, 101.9257)	15	3.24	(0.1420, 29.7340)
4	−4.58	(0.1075, −46.5715)	10	−0.27	(1.0000, 28.4632)	16	3.56	(0.1400, −78.2697)
5	−3.56	(0.0257, 65.7662)	11	0.27	(0.9706, −24.9160)	17	6.01	(0.1090, 67.4450)
6	−3.24	(0.0771, 7.0465)	12	0.73	(0.5570, −97.4171)	18	6.32	(0.1081, −58.2957)

**Table 4:** Third example: Normalized element positions and complex excitations.

*n*	$z_{n}/\lambda$	$\left(|w_{n}|\right.,\;\left.∠w_{n}\right)\;$	*n*	$z_{n}/\lambda$	$\left(|w_{n}|\right.,\;\left.∠w_{n}\right)\;$	*n*	$z_{n}/\lambda$	$\left(|w_{n}|\right.,\;\left.∠w_{n}\right)\;$
1	−3.72	(0.5171, 15.4295)	5	−1.50	(0.7644, −149.0791)	9	1.38	(0.1902, −31.2221)
2	−3.01	(1.0000, 100.9695)	6	−0.70	(0.6749, −113.2490)	10	2.12	(0.2963, −8.6059)
3	−2.53	(0.0721, 156.1117)	7	0.14	(0.4836, −77.9490)	11	2.93	(0.1309, 3.5823)
4	−2.29	(0.9066, 171.0874)	8	0.83	(0.3659, −55.6914)	12	3.75	(0.2083, 72.6957)

**Table 5:** Fourth example: Excitations.

*n*	$w_{n}$	*n*	$w_{n}$	*n*	$w_{n}$	*n*	$w_{n}$	*n*	$w_{n}$	*n*	$w_{n}$
1	−0.7703	11	3.9079	21	9.2605	31	4.3223	41	4.2946	51	3.1712
2	−0.7830	12	4.1300	22	8.3720	32	1.1883	42	1.5319	52	−0.6131
3	−1.1008	13	−1.1552	23	2.6786	33	−0.7428	43	4.2036	53	−0.5462
4	−0.9977	14	4.2489	24	−0.6591	34	3.9587	44	−1.0944	54	0.2170
5	−1.0158	15	4.1157	25	2.8896	35	5.2375	45	0.9100	55	−1.0237
6	−0.6747	16	−1.0884	26	2.1762	36	6.5611	46	3.5755	56	−1.1029
7	−0.4380	17	0.7129	27	3.1437	37	3.9997	47	3.0698	57	−0.3591
8	−0.5442	18	1.4489	28	−0.9362	38	−0.2134	48	1.0584	58	−1.0865
9	−0.9632	19	−0.7999	29	0.7927	39	9.3600	49	−0.9486	59	−1.5257
10	−1.0186	20	3.6794	30	7.0626	40	−0.1826	50	2.8547	60	−1.3586

**Table 6:** Fifth example: Excitations.

*n*	$w_{n}$	*n*	$w_{n}$	*n*	$w_{n}$	*n*	$w_{n}$	*n*	$w_{n}$	*n*	$w_{n}$
1	−0.4287	12	2.5582	23	1.6233	34	2.8681	45	1.1385	56	1.6511
2	−0.8324	13	2.3506	24	2.0073	35	0.8899	46	1.3016	57	1.7920
3	−1.3767	14	1.6307	25	2.3949	36	2.1484	47	2.2395	58	2.1606
4	0.5247	15	1.0195	26	1.5068	37	1.4631	48	2.3995	59	1.5831
5	0.7506	16	2.4243	27	1.1951	38	1.5917	49	2.8561	60	2.6363
6	1.7065	17	1.4812	28	1.9673	39	0.7089	50	1.1195	61	1.3877
7	2.0100	18	1.5143	29	2.4747	40	0.4596	51	1.4155	62	1.1193
8	1.6065	19	2.4225	30	2.6175	41	2.3680	52	1.2087
9	2.1001	20	0.5591	31	1.9543	42	1.0167	53	1.7925
10	1.3948	21	1.1460	32	3.0988	43	1.7411	54	1.6238
11	2.0795	22	2.2760	33	2.6850	44	1.1867	55	1.6902

## Abbreviations

The following abbreviations are used in this manuscript:

IoT	Internet of Things
CS	Compressive Sensing
MBR	Main Beam Region
SLR	SideLobe Region
NR	Null Region
SOCP	Second-Order Cone Program
BS	Base Station
AP	Access Point
